# Immunoproteomics and phage display in the context of leishmaniasis complexity

**DOI:** 10.3389/fimmu.2023.1112894

**Published:** 2023-02-08

**Authors:** Fernanda Ludolf, Fernanda F. Ramos, Eduardo A. F. Coelho

**Affiliations:** ^1^Programa de Pós-Graduação em Ciências da Saúde: Infectologia e Medicina Tropical, Faculdade de Medicina, Universidade Federal de Minas Gerais, Belo Horizonte, Minas Gerais, Brazil; ^2^Departamento de Patologia Clínica, Colégio Técnico (COLTEC), Universidade Federal de Minas Gerais, Belo Horizonte, Minas Gerais, Brazil

**Keywords:** *Leishmania*, biomarkers, immunoproteomics, phage display, vaccine, diagnostic

## Abstract

Leishmaniasis is defined as a complex of diseases caused by protozoan parasites of the genus *Leishmania*, which comprises 20 parasite species pathogenic to mammalians, such as humans and dogs. From a clinical point of view, and considering the diversity and biological complexity of the parasites, vectors, and vertebrate hosts, leishmaniasis is classified according to the distinct clinical manifestations, such as tegumentary (involving the cutaneous, mucosal, and cutaneous-diffuse forms) and visceral leishmaniasis. Many issues and challenges remain unaddressed, which could be attributed to the complexity and diversity of the disease. The current demand for the identification of new *Leishmania* antigenic targets for the development of multicomponent-based vaccines, as well as for the production of specific diagnostic tests, is evident. In recent years, biotechnological tools have allowed the identification of several *Leishmania* biomarkers that might potentially be used for diagnosis and have an application in vaccine development. In this Mini Review, we discuss the different aspects of this complex disease that have been addressed by technologies such as immunoproteomics and phage display. It is extremely important to be aware of the potential applications of antigens selected in different screening context, so that they can be used appropriately, so understanding their performance, characteristics, and self-limitations.

## *Leishmania* parasite complexity

The global burden of leishmaniasis has remained high for the past 10 years, with more than 220,000 new cases reported per year by the WHO (1). Immunization is the most effective way to prevent countless cases of the disease, while early diagnosis is essential for establishing an effective treatment strategy, limiting the pathology and improving the quality of life of patients. In this context, the development of new diagnostic tests and vaccines is essential for containing the disease, particularly considering that the currently available tools for disease control remain limited (2–4). In this scenario, many issues and challenges remain unaddressed due to the complexity and diversity of factors associated with this disease.

Leishmaniasis is a complex of diseases caused by more than 20 pathogenic species of protozoan parasites of the genus *Leishmania*, which are transmitted to humans through the bite of infected sandflies. The range of clinical manifestations mainly depends on the parasite species and host factors, in which the immune response plays a central role. According to the clinical manifestations, leishmaniasis can be divided into tegumentary leishmaniasis, which comprises cutaneous (CL), mucosal (ML), and cutaneous-diffuse forms (CDL), and visceral leishmaniasis (VL). CL is usually limited to an ulcer that is self-curing; however, it can evolve into severe manifestations, such as ML and CDL, which cause morbidity in patients. VL can be fatal if acute and untreated, while Post-kala-azar leishmaniasis is a skin manifestation that can occur in the populations of some countries after VL treatment (5–7).

The transmission of the parasites can occur by zoonotic or anthroponotic cycles, depending on the infecting *Leishmania* species and host background. Leishmaniasis is primarily zoonotic, with the exception of *Leishmania donovani* and *Leishmania tropica*. VL caused by *Leishmania infantum* is a zoonotic disease, in which domestic dogs are important parasite reservoirs (6, 8). Canine infection by *L. infantum* often shows a variety of cutaneous and visceral manifestations (9).

The *Leishmania* life cycle involves two main parasite forms: promastigote and amastigote. Promastigotes, the infective stage for mammalians, are inoculated into the host skin by the proboscis of the sandfly vector during the blood meal. Then, promastigotes are captured by macrophages and differentiate into amastigotes, which are the intracellular stage responsible for the development of disease (10). Leishmaniasis is a disease caused by an immunological imbalance between the host immune system and the parasite virulence factors (11).

## Leishmaniasis vaccine

Given the complexity of the disease, with many *Leishmania* species involved in different disease clinical forms, it remains unclear whether a universal vaccine or distinct vaccines are required for effective protection. The point is that, to date, a vaccine to protect against human TL and VL is not available, and the few canine vaccines available present problems and/or controversial results. The self-curing and lifelong protection that occurs in CL and in the leishmanization practice suggest that the development of a protective vaccine is feasible. However, *Leishmania* vaccinology still suffers from bottlenecks, which limit its progress (2).

Leishmanization, the century-old practice of vaccinating people with *Leishmania major* live promastigotes, was suspended by the WHO due to biosafety concerns (12, 13). Thus, developed vaccines based on crude parasite antigenic preparations achieved low efficacy in preclinical trials (2). Second-generation vaccines based on single or chimeric recombinant proteins have been tested in experimental models (14–18). However, only three *Leishmania* polyproteins composed of antigens, LEISHF1, LEISHF2, and LEISHF3, have recently entered human clinical trials (11, 19). Third-generation vaccines, based on DNA vaccines, have also been developed and evaluated in experimental models, but little progress has been made (20, 21).

So far, there are three commercially available canine leishmaniasis (CanL) vaccines: Leish-Tec1^®^, CaniLeish1^®^, and Letifend^®^. Leish-Tec1^®^ consists of the recombinant amastigote A2 protein and has shown a protective efficacy of approximately 71% in a low-infective area and 35.7% protection in a high-transmission area. The use of Leish-Tec1^®^ is problematic due to the use of the same antigen by some serological diagnostic tests, hampering the differentiation between infected and vaccinated animals (11, 22, 23). CaniLeish1^®^, which is composed of *L. infantum*-secreted/-excreted promastigote antigens, has shown variable protection results as it only decreased the infectiousness burden and the risk of dogs developing symptomatic disease (24–26). Letifend^®^ is a recombinant chimeric protein (Protein Q) that has shown 72% efficacy in preventing disease (27). In this context, owing to the variable effectivity of the few available vaccines that protect against CanL, new antigens still need to be identified and tested in experimental models and then in canines.

## Leishmaniasis immune diagnosis

Serological methods are routinely applied for the diagnosis of many infectious diseases, such as leishmaniasis. However, peculiarities of the antibody response against specific *Leishmania* antigens are crucial for the performance of serological tests. High levels of anti-*Leishmania* antibodies are usually found in patients with active VL, but they are scarce in asymptomatic cases or in those with TL, particularly CL cases. Therefore, a suitable serological test for diagnosing the distinct clinical forms of leishmaniasis is not available (28–30).

Serological diagnosis has proven to be useful in assisting the diagnosis of VL, although it is limited in terms of distinguishing between active and past or cured infections (30–33). In addition, in immunosuppressed individuals, antibody-detection tests have shown limited sensitivity (34, 35). The high molecular diversity of the rK39 homologous sequences among African *L. donovani* strains accounts for the different performances of commonly used rK39-based RDTs in different regions of the world (30). Furthermore, cross-reactivity has also been found among *Leishmania* and other diseases overlapping the same regions (36). In this context, there is still a need to identify new diagnostic antigens that can diagnose TL and improve the diagnosis of VL (28).

## Advances in biotechnological tools: Immunoproteomics and phage display

The search for new antigenic biomarkers has been ongoing for decades and has been based on empirical science, with studies evaluating soluble, crude, and/or total antigenic extracts. Nowadays, the search for more assertive and refined antigenic biomarkers can be carried out due to advances in the field of immunology, molecular biology, and high-throughput methodologies, which confer the ability to interrogate the entire antigenic repertoire and aim to improve the sensitivity and specificity of diagnostic tests, as well as the efficacy of vaccine candidates (37–41). In this context, biotechnological tools, such as immunoproteomics and phage display, have improved the scientific process and brought about the possibility of working with a tangible number of antigens, enhancing the chance of success.

Advances in proteomics have provided valuable information about proteome-wide expression profiling and host-parasite interactions, particularly with regard to virulence factors, pathogenicity, and antigenicity (39). Immunoproteomic studies, which combine proteome and serological analysis, have focused on the evaluation of immune responses in the hosts by analyzing their specific antibody/antigenic repertoire in the disease context, refining the selection of parasite antigens to be evaluated as vaccines and/or diagnostic targets (39, 42). Two-dimensional polyacrylamide gel electrophoresis (2D-PAGE) has been explored as a proteomic approach to separate the complex mixture of protein extracts into single protein spots, which is followed by mass-spectrometry and genomic/transcriptomic data analysis to identify the proteins. Up to 1,000 protein spots can be resolved on one 2D-PAGE at a time; however, owing to some limitations of the method, the profile may be restricted to highly abundant and soluble proteins. As a protein with different post-transduction modifications (PTM) migrates as distinct spots on the gel, it is not uncommon to find spots from a single gel corresponding to identical protein IDs (39, 43). The combination of 2D-PAGE and western blot (2D-WB), known as serological proteome analysis (SERPA), improves the identification of proteins recognized by antibodies present in patients´ immune sera, and such a strategy is defined as immunoproteomic (43–46).

Phage display has also been used as a biotechnological approach with the purpose of identifying new biomarkers. This is a high-throughput technology based on the selection of phage-fused surface-exposed foreign peptide variants from a clone library, which recognize/bind specific antibodies/molecules. The approach comprises an in vitro selection process in which the binding affinity assays (biopanning) using the mimotopes exposed on the phage surface are followed by DNA sequence analysis of the corresponding phage DNA. The phage display approach demonstrates the desired functionality in terms of diagnosis and vaccine development (47, 48).

## Biotechnological tools and their application for leishmaniasis

Comparative analyses of sequenced and annotated *Leishmania* genomes have uncovered little variation in unique gene content across parasite species. Nonetheless, large-scale genetic differences in terms of gene and chromosome copy number have been found to contribute to alterations in gene expression in response to environmental conditions in mammalian hosts (49). To figure out the functional differences of proteins among species, proteomic studies have been conducted to compare the promastigote and amastigote stages using total and/or secreted protein extracts, with results showing some modest differences in protein expression levels (50).

*Leishmania* immunoreactive proteins have been identified through the association of high-throughput and serology methodologies, such as immunoproteomics and phage display, when sera samples from infected canine and human patients were used in the protocols. Several *Leishmania* immunoproteomic and phage display studies have been conducted, each of them trying to answer specific questions regarding the complexity of the disease (**Tables 1**, **2**). Several proteins have been identified as antigenic, in addition to antigens already described in the literature, along with a large number of hypothetical proteins that, together, represent an important contribution (66, 67). With these studies, antigens with immunogenic and/or serological diagnostic potential with human and canine disease were identified in the context of the complexity of the disease. In this review, these topics are addressed.

**Table 1:** Summary of *Leishmania* immunoproteomic studies.

Clinical manifestation	Species/strain	Parasite stage	Sample	Criteria of exclusions	Number of identified proteins using MS	Reference
TL	*L. braziliensis* (MHOM/BR/1975/ M2904)	Promastigote Amastigote-like	- Brazilian ML sera - Brazilian CL sera	- Spots reactive to endemic area non-infected subject sera - Spots reactive to Chagas disease patient sera	- 6 for promastigote - 4 for amastigote - 10 for both stages	Duarte et al. (51)
TL	*L. amazonensis* (IFLA/BR/1967/PH8) *L. braziliensis* (MHOM/ BR/1975/M2904)	Promastigote	Brazilian TL sera	- Spots reactive to healthy subject sera - Spots reactive to HVL sera - Spots from *L. infantum* reactive to TL sera - Low abundant immunoreactive spots	- 9 for *L. amazonesis* - 2 for *L. braziliensis*	Lima et al. (52)
HVL	*L. infantum* (MHOM/BR/1972/BH46)	Promastigote	Brazilian HVL sera	- Spots reactive to healthy subject sera - Spots reactive to TL sera - Spots from *L. amazonensis* and *L. braziliensis* reactive to VL sera - Low abundant immunoreactive spots	9	Lima et al. (52)
HVL	*L. infantum* (MHOM/BR/1970/BH46)	- Promastigote - Amastigote-like	Brazilian HVL sera	- Spots reactive to endemic area non-infected subject sera - Spots reactive to Chagas disease patient sera	- 29 for promastigote stage - 21 for amastigote stage	Lage at al. (53).
HVL	*L. infantum* (MCAN/IR/14/M14)	Promastigote	Iranian HVL sera	Spots reactive to healthy subject sera	11	Heidari et al. (54)
HVL	*L. infantum* (MHOM/BR/1970/BH46)	Promastigote	- Brazilian HLV/HIV sera - Brazilian HLV sera	Spots reactive to HIV positive sera not co-infected with LV	26 for both VL and VL/HIV sera	Machado et al. (55)
HVL	*L. donovani* (MHOM/IN/02/BHU2, MHOM/IN/02/BHU17, and AG83)	Promastigote	Indian HLV sera	Spots not reactive to all 4 individually sera	6	Forgber et al. (56)
HVL	*L. donovani* (AG83)	Promastigote	Indian HLV urine	- Bands reactive to healthy subject sera - Bands reactive to malaria, viral fever, tuberculosis, Typhoid, and pneumonia sera	3	Ejazi et al. (57)
HVL	*- Leishmania chagasi* (MHOM/BR00/MER/STRAIN2)	Promastigote	Brazilian HVL sera		- 2 for VL pretreatment - 5 for VL post-treatment - 5 for VL treatment resistant/asymptomatic - 2 for all	Abanades et al. (58)

MS, Mass Spectrometry.

**Table 2:** Summary of *Leishmania* phage display studies.

Clinical manifestation	Species/strain	Phage display (library)	Sample (positive selection)	Sample (negative selection)	Number of phage clones selected by phage display	Number of non-repetitive peptides on selected phage clones	Reference
CVL	*L. infantum*	M13 filamentous phages C7C library	Brazilian CVL sera	- Endemic area non-infected dog sera - *Trypanossoma cruzi*-infected dogs sera	96 phage clones	18	Costa et al. (59)
CVL	*L. infantum*	M13 phage (X15) and (XCX 8CX) libraries	Brazilian CVL sera	- Endemic area non-infected dogssera - *T. cruzi*-infected dogs sera	198 phage clones 12 clones (abs ≥ 1.0)	n/a	Toledo-Machado et al. (60)
HVL	*L. infantum*	M13 filamentous phages C7C library	Brazilian HVL sera	- Endemic area non-infected human sera - *T.cruzi*-infected Human sera	96 phage clones 42 phage clones (abs> SLA)	8	Salles et al. (61)
HVL	*L. infantum*	M13 filamentous phages C7C library	Brazilian HVL sera	- Endemic area non-infected human sera - *T.cruzi*-infected Human sera	76 phage clones	17	Ramos et al. (62)
HVL	*L. infantum*	M13 filamentous phages C7C library	Brazilian HVL/HIV sera	- Endemic area non-infected human sera - HIV-infected human sera	48 phage clones	9	Ramos et al. (63)
TL	*L. braziliensis*	M13 phage (LX4), (LX8), (X15), and (X8CX8) libraries	Brazilian CL sera	Endemic area non-infected human sera	428 phage clones 36 phage clones (abs> 0.5 - SLA)	3	Link et al. (64)
TL	*L. amazonensis*	M13 filamentous phages C7C library	Brazilian TL sera	Endemic area non-infected human sera	58 phage clones	9	Carvalho et al. (65)

Abs, absorbance at 492 nm; SLA, soluble leishmania antigen.

## Immunoproteomics for tegumentary leishmaniasis

To solve problems associated with the serodiagnosis of TL (low levels of antileishmanial antibodies are usually found in most patients), an immunoproteomic approach involving 2D-PAGE of *Leishmania braziliensis* total protein extracts and sera from ML and CL patients was conducted. This study identified 20 potential candidates, which were specific to antibodies in the sera (51). Six of these identified proteins were found only at the promastigote stage, four at the amastigote-like stage, and 10 at both parasite stages. An ELISA evaluation of the tryparedoxin peroxidase recombinant protein, which was identified in this study, showed 100% sensitivity and specificity for both CL and ML (51). Proteins found in this immunoproteomic study, such as enolase protein, eukaryotic elongation factor-1 beta protein, eukaryotic Initiation factor 5a, tryparedoxin peroxidase, and hypothetical protein LbHyM, were later evaluated and showed high potential for the serodiagnosis of the disease or immunogenic efficacy against TL and/or even VL in a murine model (18, 68–72).

In another study, Lima et al. (52) conducted a 2D-WB using total protein extracts from *L. amazonensis, L. braziliensis*, and *L. infantum* against sera pools of TL or VL patients. To identify biomarkers specific to TL, spots from *L. amazonensis* and *L. braziliensis* reactive to VL sera and spots from *L. infantum* reactive to TL sera were all excluded. MS analysis identified nine proteins for *L. amazonesis* and two proteins for *L. braziliensis*, which were considered abundant and immunoreactive against TL sera (52). These antigens may help the development of a specific diagnostic test for TL, distinguishing it from the VL form.

## Immunoproteomics for visceral leishmaniasis

An immunoproteomic approach performed with *L. infantum* protein extract, promastigotes, and amastigotes in reactions against sera from Brazilian HVL patients identified 29 promastigote proteins and 21 amastigote proteins. The authors suggested that such antigens could be evaluated as diagnostic or vaccine candidates against the disease (53). Two of these identified antigens, endonuclease III and GTP-binding protein, were expressed and tested in an ELISA against a serological panel, and results showed around 100% sensitivity and specificity for HVL diagnosis (53). The proteins rGTP and pyridoxal kinase and the hypothetical proteins LiHyQ and LiHyJ, identified in this immunoproteomic study, were evaluated afterwards and showed potential to be developed as a new serodiagnostic test and/or a vaccine (21, 73–76). LiHyQ was evaluated for its ability to diagnose TL, HVL and CanVL and showed a superior diagnostic performance compared with a parasite antigenic preparation and two commercial kits (73). Additionally, LiHyJ showed high sensitivity and specificity values in diagnosing HVL and canVL (74). Furthermore, rGTP protein was recently shown to be protective against experimental VL in a murine model (76). LyHyJ and pyridoxal kinase, administered either as a DNA plasmid or recombinant protein, showed potential as immunogens against HVL (21, 75).

Abánades et al. (58) conducted a *Leishmania chagasi* 2D-WB to investigate issues regarding the low antibody titers in asymptomatic responders and the poorly characterized serologic markers of recovery or resistance to infection. This study identified two antigens exclusively for VL pretreatment, five for VL post-treatment, five for VL treatment in resistant/asymptomatic patients, and two that were common to all.

An immunoproteomic study using *L. infantum* promastigote Iranian strain and pooled sera of Iran VL endemic area subjects was conducted, and 11 immunoreactive proteins were identified. A recombinant multi-epitope antigen was designed from the list of identified proteins and evaluated for its potential performance in the serodiagnosis of human VL (54).

As coinfection between VL and human immunodeficiency virus (HIV) has increased in several countries and current serological tests are not sensitive enough to detect most VL/HIV cases, new studies are needed to identify more sensitive antigens. In this context, Machado et al. (55) developed an immunoproteomic approach to search for new biomarkers that could have the capacity to diagnose VL and VL/HIV co-infection cases. Results showed that 43 protein spots were recognized by antibodies in both VL and VL/HIV sera, and 26 proteins were identified by mass spectrometry. One of them, β-tubulin, was expressed, purified, and evaluated using an ELISA as a proof-of-concept for validating their findings, and results showed significant sensitivity and specificity values in diagnosing both VL and VL/HIV patients.

Silver-stained 2D-PAGE of *L. donovani* crude extract revealed 1,067 protein spots, while corresponding 2D-WB identified 330 antigens. From those, 68 antigens could be assigned to the stained spots, showing that the antigenicity of these proteins did not correlate with protein expression levels. Six proteins were identified by MS. Western blot of *L. donovani* incubated individually with sera from 15 VL patients showed bands with a broad range of immune specificities and extensive heterogeneity of the serological response (56).

A urine-based ELISA has been suggested as a non-invasive, simple, and safe alternative for diagnosing infectious diseases, such as VL (77, 78). Urine antibodies against 51, 55, and 63 kDa proteins were found with 100% recognition by samples from VL patients. Spots with the same molecular weight size were excised from 2D-PAGE and the proteins were identified as elongation factor 1α of *L. infantum*, α-tubulin of *L. donovani*, and glycoprotein or leishmanolysin of *L. donovani*, respectively (57).

## Immunoproteomics for canine leishmaniasis

Dogs are the main domestic reservoir of *Leishmania* and, as such, are important in the maintenance of the transmission cycle. As the occurrence of asymptomatic infection is considerably higher than that of apparent clinical illness in the infected animals, it is very important to have a more efficient diagnostic test. Additionally, the detection of antigens in asymptomatic dogs could lead to the identification of more assertive vaccine candidates. Machado et al. (79) reviewed the advances in CanL antigen identification using immunoproteomics and phage display and explored the results obtained with these technologies and the validation of some of the identified hypothetical proteins. Briefly, to date, immunoproteomic approaches have been performed with protein extracts from the amastigotes and promastigotes of *L. infantum* and *L. chagasi* in reactions against sera of asymptomatic and symptomatic VL dogs from different regions of the world (14, 42, 80, 81).

## Phage display in *Leishmania*

Phage display has been used to identify *Leishmania* mimotopes that may have a potential application as diagnostic markers and vaccine candidates against leishmaniasis (**Table 2**). In a phage display study, Costa et al. (59) identified eighteen mimotopes, eight of which showed approximately 100% sensitivity and specificity in an ELISA performed against canine VL sera. Two mimotopes, as well as their synthetic peptides, were later tested for the diagnosis of human VL, and results showed also significant sensitivity and specificity values (82). Additionally, phage display technology was used to select clones of phage-exposed peptide-specific antibodies from asymptomatic and symptomatic VL patients. Eight phage clones were selected after the biopanning cycles, and their reactivity was evaluated using a phage-ELISA, with results showing they were highly efficacious at identifying VL patients, thus demonstrating the feasibility of using mimotopes for the development of a more specific and sensitive serodiagnosis of VL (61). Sera from *L. infantum*-infected dogs were used in another phage display biopanning, and the corresponding peptides were evaluated as vaccine candidates in a murine model, with aluminum hydroxide and a liposome formulation eliciting significant protection (60). Selected phage clones reactive to HVL sera and with a Th1 direction after stimulus of human peripheral blood mononuclear cells (PBMCs) were also used to immunize BALB/c mice, and a partial protection against the parasite challenge was observed (62). Carvalho et al. (65) used the same experimental strategy, in which sera of TL patients were used to select specific mimotopes, followed by immune stimulation of human PBMCs. Two selected clones were then tested in immunization protocols in BALB/c mice, and protection against *L. amazonensis* infection was achieved, with significant reductions in the parasite load and footpad swelling. In another study, Ramos et al. (63) selected nine mimotopes using sera from VL and VL/HIV patients in the biopanning cycles. The corresponding epitopes were produced as synthetic peptides and evaluated using an ELISA against a human serological panel. Three of these antigens were able to diagnose both VL and VL/HIV coinfection with approximately 100% efficacy. Three peptides were synthetized after their mimotopes where identified by phage display using antibodies from *L. braziliensis* patients. Antibodies produced in hamsters immunized with these peptides reacted with bands of different sizes in SDS-PAGE with *L. braziliensis* protein extract. Additionally, peptides were recognized by sera from CL patients in an ELISA (64).

## Conclusion

With the applicability of biotechnological tools such as immunoproteomics and phage display, several antigens have been identified in different contexts of the complexity of leishmaniasis. Immunoproteomics and phage display studies are characterized by the intention of opening doors to new studies more than generating final conclusions, and in this way, the specific outcomes or identified biomarkers fall into a list of proteins/mimotopes that could be assessed in future original studies. Such strategies have showed applicability in this selection process but future studies validating their findings in target mammalian models are certainly necessary to define the protective or diagnostic status of these antigens for canine and human leishmaniasis. Some of them have been successfully evaluated in experimental trials as vaccine or diagnostic candidates. The current demand for new *Leishmania* antigens for the development of multicomponent vaccines and for the production of specific diagnostic tests is evident, and these processes have been aided by immunoproteomic and phage-display approaches. It is very important to be aware of the objective of screening antigens so that they are used properly and their performance, characteristics, and limitations are well understood.

## Author contributions

FL designed the manuscript. FL, FFR, and EAFC wrote the manuscript. FL and EAFC revised the manuscript. All authors contributed to the article and approved the submitted version.

## References

[1] World Health Organization. Global leishmaniasis update, 2006–2015: A turning point in leishmaniasis surveillance (2017). Available at: https://www.who.int/publications/i/item/who-wer9238.

[2] Schroeder J Aebischer T. Vaccines for leishmaniasis: From proteome to vaccine candidates. Hum Vaccin (2011) 7(Suppl):10–5. doi: 10.4161/hv.7.0.14556 21245661

[3] Sundar S Singh OP Chakravarty J. Visceral leishmaniasis elimination targets in India, strategies for preventing resurgence. Expert Rev Anti Infect Ther (2018) 16:805–12. doi: 10.1080/14787210.2018.1532790 PMC6345646 30289007

[4] Mann S Frasca K Scherrer S Henao-Martínez AF Newman S Ramanan P. A review of leishmaniasis: Current knowledge and future directions. Curr Trop Med Rep (2021) 8(2):121–32. doi: 10.1007/s40475-021-00232-7 PMC7966913 33747716

[5] Colmenares M Kar S Goldsmith-Pestana K McMahon-Pratt D. Mechanisms of pathogenesis: differences amongst leishmania species. Trans R Soc Trop Med Hyg (2002) 96 Suppl 1:S3–7. doi: 10.1016/s0035-9203(02)90044-1 12055848

[6] Burza S Croft SL Boelaert M. Leishmaniasis. Lancet (2018) 392(10151):951–70. doi: 10.1016/S0140-6736(18)31204-2 30126638

[7] Al-Khalaifah HS. Major molecular factors related to leishmania pathogenicity. Front Immunol (2022) 13:847797. doi: 10.3389/fimmu.2022.847797 35769465 PMC9236557

[8] Quinnell RJ Courtenay O. Transmission, reservoir hosts and control of zoonotic visceral leishmaniasis. Parasitology (2009) 136(14):1915–34. doi: 10.1017/S0031182009991156 19835643

[9] Abbehusen MMC Almeida VDA Solcà MDS Pereira LDS Costa DJ Gil-Santana L. Clinical and immunopathological findings during long term follow-up in leishmania infantum experimentally infected dogs. Sci Rep (2017) 7(1):15914. doi: 10.1038/s41598-017-15651-8 29162847 PMC5698407

[10] Kaye P Scott P. Leishmaniasis: Complexity at the host-pathogen interface. Nat Rev Microbiol (2011) 9(8):604–15. doi: 10.1038/nrmicro2608 21747391

[11] Iborra S Solana JC Requena JM Soto M. Vaccine candidates against leishmania under current research. Expert Rev Vaccines (2018) 17(4):323–34. doi: 10.1080/14760584.2018.1459191 29589966

[12] Mohebali M Nadim A Khamesipour A. An overview of leishmanization experience: A successful control measure and a tool to evaluate candidate vaccines. Acta Trop (2019) 200:105173. doi: 10.1016/j.actatropica.2019.105173 31525323

[13] Solana JC Moreno J Iborra S Soto M Requena JM. Live attenuated vaccines, a favorable strategy to provide long-term immunity against protozoan diseases. Trends Parasitol (2022) 38(4):316–34. doi: 10.1016/j.pt.2021.11.004 34896016

[14] Coelho VT Oliveira JS Valadares DG Chávez-Fumagalli MA Duarte MC Lage PS. Identification of proteins in promastigote and amastigote-like leishmania using an immunoproteomic approach. PloS Negl Trop Dis (2012) 6(1):e1430. doi: 10.1371/journal.pntd.0001430 22272364 PMC3260309

[15] Martins VT Chávez-Fumagalli MA Costa LE Canavaci AM Lage PS Lage DP. Correction: Antigenicity and protective efficacy of a leishmania amastigote-specific protein, member of the super-oxygenase family, against visceral leishmaniasis. PloS Negl Trop Dis (2013) 7(3): e2148. doi: 10.1371/annotation/427655ac-c278-41f5-95ca-6279c562752f 23573301 PMC3610918

[16] Costa LE Goulart LR Pereira NC Lima MI Duarte MC Martins VT. Mimotope-based vaccines of leishmania infantum antigens and their protective efficacy against visceral leishmaniasis. PloS One (2014) 9(10):e110014. doi: 10.1371/journal.pone.0110014 25333662 PMC4198211

[17] Duarte MC Lage DP Martins VT Chávez-Fumagalli MA Roatt BM Menezes-Souza D. Recent updates and perspectives on approaches for the development of vaccines against visceral leishmaniasis. Rev Soc Bras Med Trop (2016) 49(4):398–407. doi: 10.1590/0037-8682-0120-2016 27598624

[18] Duarte MC Lage DP Martins VT Costa LE Carvalho AMRS Ludolf F. A vaccine composed of a hypothetical protein and the eukaryotic initiation factor 5a from leishmania braziliensis cross-protection against leishmania amazonensis infection. Immunobiology (2017) 222(2):251–60. doi: 10.1016/j.imbio.2016.09.015 27693018

[19] Duthie MS Reed SG. Not all antigens are created equally: Progress, challenges, and lessons associated with developing a vaccine for leishmaniasis. Clin Vaccine Immunol (2017) 24:1–7.e00108–17. doi: 10.1128/CVI.00108-17 PMC5498718 28515135

[20] Ribeiro PAF Dias DS Lage DP Martins VT Costa LE Santos TTO. Immunogenicity and protective efficacy of a new leishmania hypothetical protein applied as a DNA vaccine or in a recombinant form against leishmania infantum infection. Mol Immunol (2019) 106:108–18. doi: 10.1016/j.molimm.2018.12.025 30594673

[21] Oliveira-da-Silva JA Lage DP Ramos FF Machado AS Tavares GSV Mendonça DVC. Corrigendum to "Leishmania infantum pyridoxal kinase evaluated in a recombinant protein and DNA vaccine to protects against visceral leishmaniasis". Mol Immunol (2020) 124:161–71. doi: 10.1016/j.molimm.2020.09.002 32585510

[22] Fernandes AP Costa MM Coelho EA Michalick MS de Freitas E Melo MN. Protective immunity against challenge with leishmania (Leishmania) chagasi in beagle dogs vaccinated with recombinant A2 protein. Vaccine (2008) 26(46):5888–95. doi: 10.1016/j.vaccine.2008.05.095 18786587

[23] Grimaldi G Teva A Dos-Santos CB Santos FN Pinto ID Fux B. Field trial of efficacy of the leish-tec® vaccine against canine leishmaniasis caused by leishmania infantum in an endemic area with high transmission rates. PloS One (2017) 12:e0185438. doi: 10.1371/journal.pone.0185438 28953944 PMC5617193

[24] Moreno J Vouldoukis I Martin V McGahie D Cuisinier AM Gueguen S. Use of a LiESP/QA-21 vaccine (CaniLeish) stimulates an appropriate Th1-dominated cell-mediated immune response in dogs. PloS Negl Trop Dis (2012) 6(6):e1683. doi: 10.1371/journal.pntd.0001683 22724031 PMC3378610

[25] Oliva G Nieto J Foglia Manzillo V Cappiello S Fiorentino E Di Muccio T. A randomised, double-blind, controlled efficacy trial of the LiESP/QA-21 vaccine in naïve dogs exposed to two leishmania infantum transmission seasons [published correction appears in PLoS negl trop dis. 2014 Nov;8(11):e3408]. PloS Negl Trop Dis (2014) 8(10):e3213. doi: 10.1371/journal.pntd.0003213 25299614 PMC4191955

[26] Martin V Vouldoukis I Moreno J McGahie D Gueguen S Cuisinier AM. The protective immune response produced in dogs after primary vaccination with the LiESP/QA-21 vaccine (CaniLeish®) remains effective against an experimental challenge one year later. Vet Res (2014) 45(1):69. doi: 10.1186/1297-9716-45-69 24964736 PMC4086268

[27] Fernández Cotrina J Iniesta V Monroy I Baz V Hugnet C Marañon F Fabra M. A large-scale field randomized trial demonstrates safety and efficacy of the vaccine LetiFend® against canine leishmaniosis. Vaccine (2018) 36(15):1972–82. doi: 10.1016/j.vaccine.2018.02.111 29525281

[28] WHO. Target product profile for a point-of-care diagnostic test for dermal leishmaniases (2022). Available at: https://www.who.int/publications/i/item/9789240045224. 10.1016/j.parepi.2019.e00103 PMC6423987 30923755

[29] de Vries HJC Reedijk SH Schallig HDFH. Cutaneous leishmaniasis: recent developments in diagnosis and management. Am J Clin Dermatol (2015) 16:99–109. doi: 10.1007/s40257-015-0114-z 25687688 PMC4363483

[30] Kaye PM Cruz I Picado A Van Bocxlaer K Croft SL. Leishmaniasis immunopathology–impact on design and use of vaccines, diagnostics and drugs. Semin Immunopathol (2020) 42:247–64. doi: 10.1007/s00281-020-00788-y 32152715

[31] Sundar S Rai M. Laboratory diagnosis of visceral leishmaniasis. Clin Diagn Lab Immunol (2002) 9(5):951–8. doi: 10.1128/cdli.9.5.951-958.2002 PMC120052 12204943

[32] Sundar S Sahu M Mehta H Gupta A Kohli U Rai M. Noninvasive management of Indian visceral leishmaniasis: clinical application of diagnosis by K39 antigen strip testing at a kala-azar referral unit. Clin Infect Dis (2002) 35(5):581–6. doi: 10.1086/342057 12173133

[33] Gidwani K Picado A Ostyn B Singh SP Kumar R Khanal B. Persistence of leishmania donovani antibodies in past visceral leishmaniasis cases in India. Clin Vaccine Immunol (2011) 18(2):346–8. doi: 10.1128/CVI.00473-10 PMC3067357 21159922

[34] Cota GF de Sousa MR Demarqui FN Rabello A. The diagnostic accuracy of serologic and molecular methods for detecting visceral leishmaniasis in HIV infected patients: meta-analysis. PloS Negl Trop Dis (2012) 6:e1665. doi: 10.1371/journal.pntd.0001665 22666514 PMC3362615

[35] Lindoso JAL Moreira CHV Cunha MA Queiroz IT. Visceral leishmaniasis and HIV coinfection: current perspectives. HIV AIDS. (2018) 10:193–201. doi: 10.2147/HIV.S143929 PMC6197215 30410407

[36] Kohanteb J Ardehali S. Cross-reaction of sera from patients with various infectious diseases with leishmania infantum. Med Princ Pract (2005) 14:79–82. doi: 10.1159/000083915 15785097

[37] Rappuoli R. Reverse vaccinology. Curr Opin Microbiol (2000) 3(5):445–50. doi: 10.1016/s1369-5274(00)00119-3 11050440

[38] Moxon R Reche PA Rappuoli R. Editorial: Reverse vaccinology. Front Immunol (2019) 10:2776. doi: 10.3389/fimmu.2019.02776 31849959 PMC6901788

[39] Aslam B Basit M Nisar MA Khurshid M Rasool MH. Proteomics: Technologies and their applications. J Chromatogr Sci (2017) 55(2):182–96. doi: 10.1093/chromsci/bmw167 28087761

[40] Seib KL Zhao X Rappuoli R. Developing vaccines in the era of genomics: A decade of reverse vaccinology. Clin Microbiol Infect (2012) 18 Suppl 5:109–16. doi: 10.1111/j.1469-0691.2012.03939.x 22882709

[41] Rinaudo CD Telford JL Rappuoli R Seib KL. Vaccinology in the genome era. J Clin Invest. (2009) 119(9):2515–25. doi: 10.1172/JCI38330 PMC2735939 19729849

[42] Rashidi S Kalantar K Hatam G. Using proteomics as a powerful tool to develop a vaccine against Mediterranean visceral leishmaniasis. J Parasit Dis (2018) 42(2):162–70. doi: 10.1007/s12639-018-0986-y PMC5962495 29844618

[43] Rabilloud T Lelong C. Two-dimensional gel electrophoresis in proteomics: a tutorial. J Proteomics. (2011) 74(10):1829–41. doi: 10.1016/j.jprot.2011.05.040 21669304

[44] Klade CS. Proteomics approaches towards antigen discovery and vaccine development. Curr Opin Mol Ther (2002) 4(3):216–23. 12139306

[45] Fulton KM Ananchenko A Wolfraim L Martin S Twine SM. Classical immunoproteomics: Serological proteome analysis (SERPA) for antigen identification. Immunoproteomics (2019), 2024(2019) 59–78. doi: 10.1007/978-1-4939-9597-4_3 31364042

[46] Purcell AW Gorman JJ. Immunoproteomics: Mass spectrometry-based methods to study the targets of the immune response. Mol Cell Proteomics MCP (2004) 3(3):193–208. doi: 10.1074/mcp.R300013-MCP200 14718575

[47] Goulart LR Vieira CU Freschi AP Capparelli FE Fujimura PT Almeida JF. Biomarkers for serum diagnosis of infectious diseases and their potential application in novel sensor platforms. Crit Rev Immunol (2010) 30(2):201–22. doi: 10.1615/critrevimmunol.v30.i2.70 20370630

[48] Smith GP Petrenko VA. Phage display. Chem Rev (1997) 97(2):391–410. doi: 10.1021/cr960065d 11848876

[49] Rogers MB Hilley JD Dickens NJ Wilkes J Bates PA Depledge DP. Chromosome and gene copy number variation allow major structural change between species and strains of leishmania. Genome Res (2011) 21(12):2129–42. doi: 10.1101/gr.122945.111 PMC3227102 22038252

[50] Pissarra J Pagniez J Petitdidier E Séveno M Vigy O Bras-Gonçalves R. Proteomic analysis of the promastigote secretome of seven leishmania species. J Proteome Res (2022) 21(1):30–48. doi: 10.1021/acs.jproteome.1c00244 34806897

[51] Duarte MC Pimenta DC Menezes-Souza D Magalhães RD Diniz JL Costa LE]. Proteins selected in leishmania (Viannia) braziliensis by an immunoproteomic approach with potential serodiagnosis applications for tegumentary leishmaniasis. Clin Vaccine Immunol (2015) 22(11):1187–96. doi: 10.1128/CVI.00465-15 PMC4622107 26376929

[52] Lima BS Pires SF Fialho LC Jr Oliveira EJ Machado-de-Avila RA Chávez-Olórtegui C. A proteomic road to acquire an accurate serological diagnosis for human tegumentary leishmaniasis. J Proteomics. (2017) 151:174–81. doi: 10.1016/j.jprot.2016.05.017 27262223

[53] Lage DP Ludolf F Silveira PC Machado AS Ramos FF Dias DS. Screening diagnostic candidates from leishmania infantum proteins for human visceral leishmaniasis using an immunoproteomics approach. Parasitology (2019) 146(11):1467–76. doi: 10.1017/S0031182019000714 31142384

[54] Heidari S Hajjaran H Kazemi B Gharechahi J Mohebali M Ranjbar MM. Identification of immunodominant proteins of leishmania infantum by immunoproteomics to evaluate a recombinant multi-epitope designed antigen for serodiagnosis of human visceral leishmaniasis. Exp Parasitol (2021) 222:108065. doi: 10.1016/j.exppara.2021.108065 33428893

[55] Machado AS Ramos FF Oliveira-da-Silva JA Santos TTO Tavares GSV Costa LE. An immunoproteomics approach to identify leishmania infantum proteins to be applied for the diagnosis of visceral leishmaniasis and human immunodeficiency virus co-infection. Parasitology (2020) 147(9):932–9. doi: 10.1017/S0031182020000578 PMC10317630 32308186

[56] Forgber M Basu R Roychoudhury K Theinert S Roy S Sundar S. Mapping the antigenicity of the parasites in leishmania donovani infection by proteome serology. PloS One (2006) 1(1):e40. doi: 10.1371/journal.pone.0000040 17183669 PMC1762392

[57] Ejazi SA Bhattacharyya A Choudhury ST Ghosh S Sabur A Pandey K. Immunoproteomic identification and characterization of leishmania membrane proteins as non-invasive diagnostic candidates for clinical visceral leishmaniasis. Sci Rep (2018) 8(1):12110. doi: 10.1038/s41598-018-30546-y 30108316 PMC6092337

[58] Abánades DR Arruda LV Arruda ES Pinto JR Palma MS Aquino D. Immunodominant antigens of leishmania chagasi associated with protection against human visceral leishmaniasis. PloS Negl Trop Dis (2012) 6(6):e1687. doi: 10.1371/journal.pntd.0001687 22724032 PMC3378602

[59] Costa LE Lima MI Chávez-Fumagalli MA Menezes-Souza D Martins VT Duarte MC. Subtractive phage display selection from canine visceral leishmaniasis identifies novel epitopes that mimic leishmania infantum antigens with potential serodiagnosis applications. Clin Vaccine Immunol (2014) 21(1):96–106. doi: 10.1128/CVI.00583-13 24256622 PMC3910914

[60] Toledo-Machado CM Bueno LL Menezes-Souza D Machado-de-Avila RA Nguyen C Granier C. Use of phage display technology in development of canine visceral leishmaniasis vaccine using synthetic peptide trapped in sphingomyelin/cholesterol liposomes. Parasit Vectors. (2015) 8:133. doi: 10.1186/s13071-015-0747-z 25889286 PMC4352561

[61] Salles BC Costa LE Alves PT Dias AC Vaz ER Menezes-Souza D. Leishmania infantum mimotopes and a phage-ELISA assay as tools for a sensitive and specific serodiagnosis of human visceral leishmaniasis. Diagn Microbiol Infect Dis (2017) 87(3):219–25. doi: 10.1016/j.diagmicrobio.2016.11.012 27939286

[62] Ramos FF Costa LE Dias DS Santos TTO Rodrigues MR Lage DP. Selection strategy of phage-displayed immunogens based on an *in vitro* evaluation of the Th1 response of PBMCs and their potential use as a vaccine against leishmania infantum infection. Parasit Vectors. (2017) 10(1):617. doi: 10.1186/s13071-017-2576-8 29268793 PMC5740923

[63] Ramos FF Tavares GSV Ludolf F Machado AS Santos TTO Gonçalves IAP. Diagnostic application of sensitive and specific phage-exposed epitopes for visceral leishmaniasis and human immunodeficiency virus coinfection. Parasitology (2021) 148(13):1706–14. doi: 10.1017/S0031182021001505 PMC11010164 35060464

[64] Link JS Alban SM Soccol CR Pereira GVM Thomaz Soccol V. Synthetic peptides as potential antigens for cutaneous leishmaniosis diagnosis. J Immunol Res (2017) 2017:5871043. doi: 10.1016/j.micpath.2020.104283 28367456 PMC5359444

[65] Carvalho GB Costa LE Lage DP Ramos FF Santos TTO Ribeiro PAF]. High-through identification of T cell-specific phage-exposed mimotopes using PBMCs from tegumentary leishmaniasis patients and their use as vaccine candidates against leishmania amazonensis infection. Parasitology (2019) 146(3):322–32. doi: 10.1017/S0031182018001403 30198459

[66] Kumari S Kumar A Samant M Sundar S Singh N Dube A. Proteomic approaches for discovery of new targets for vaccine and therapeutics against visceral leishmaniasis. Proteomics Clin Appl (2008) 2(3):372–86. doi: 10.1002/prca.200780017 21136840

[67] Magalhães RD Duarte MC Mattos EC Martins VT Lage PS Chávez-Fumagalli MA. Identification of differentially expressed proteins from leishmania amazonensis associated with the loss of virulence of the parasites. PloS Negl Trop Dis (2014) 8(4):e2764. doi: 10.1371/journal.pntd.0002764 24699271 PMC3974679

[68] Lima MP Costa LE Duarte MC Menezes-Souza D Salles BCS de Oliveira Santos TT. Evaluation of a hypothetical protein for serodiagnosis and as a potential marker for post-treatment serological evaluation of tegumentary leishmaniasis patients. Parasitol Res (2017) 116(4):1197–206. doi: 10.1007/s00436-017-5397-y 28150041

[69] Duarte MC Lage DP Martins VT Costa LE Salles BCS Carvalho AMRS. Performance of leishmania braziliensis enolase protein for the serodiagnosis of canine and human visceral leishmaniosis. Vet Parasitol (2017) 238:77–81. doi: 10.1016/j.vetpar.2017.03.024 28385540

[70] Santos TTO Martins VT Lage DP Costa LE Salles BCS Carvalho AMRS. Probing the efficacy of a heterologous Leishmania/L. viannia braziliensis recombinant enolase as a candidate vaccine to restrict the development of l. infantum in BALB/c mice. Acta Trop (2017) 171:8–16. doi: 10.1016/j.actatropica.2017.03.008 28288798

[71] Santos TTO Cardoso MS Machado AS Siqueira WF Ramos FF Oliveira-da-Silva JA. Recombinant leishmania eukaryotic elongation factor-1 beta protein: A potential diagnostic antigen to detect tegumentary and visceral leishmaniasis in dogs and humans. Microb Pathog (2019) 137:103783. doi: 10.1016/j.micpath.2019.103783 31600536

[72] Medeiros RMTE Carvalho AMRS Ferraz IA Medeiros FAC Cruz LDR Rocha MODC. Mapping linear b-cell epitopes of the tryparedoxin peroxidase and its implications in the serological diagnosis of tegumentary leishmaniasis. Acta Trop (2022) 232:106521. doi: 10.1016/j.actatropica.2022.106521 35595092

[73] Santos TTO Ramos FF Gonçalves IAP Tavares GSV Ludolf F Bandeira RS. Potential of recombinant LiHyQ, a novel leishmania infantum protein, for the diagnosis of canine visceral leishmaniasis and as a diagnostic and prognostic marker for human leishmaniasis and human immunodeficiency virus co-infection: A preliminary study. Acta Trop (2021) 224:106126. doi: 10.1016/j.actatropica.2021.106126 34537185

[74] Oliveira-da-Silva JA Machado AS Tavares GSV Ramos FF Lage DP Ludolf F. Biotechnological applications from a leishmania amastigote-specific hypothetical protein in the canine and human visceral leishmaniasis. Microb Pathog (2020) 147:104283. doi: 10.1016/j.micpath.2020.104283 32485231

[75] Oliveira-da-Silva JA Machado AS Ramos FF Tavares GSV Lage DP Mendonça DVC. A leishmania amastigote-specific hypothetical protein evaluated as recombinant protein plus Th1 adjuvant or DNA plasmid-based vaccine to protect against visceral leishmaniasis. Cell Immunol (2020) 356:104194. doi: 10.1016/j.cellimm.2020.104194 32827943

[76] Lage DP Machado AS Vale DL Freitas CS Linhares FP Cardoso JMO. Recombinant guanosine-5'-triphosphate (GTP)-binding protein associated with poloxamer 407-based polymeric micelles protects against leishmania infantum infection. Cytokine (2022) 153:155865. doi: 10.1016/j.cyto.2022.155865 35339043

[77] Nagaoka F Yamazaki T Akashi-Takamura S Itoh M. Detection of urinary antibodies and its application in epidemiological studies for parasitic diseases. Vaccines (Basel). (2021) 9(7):778. doi: 10.3390/vaccines9070778 34358194 PMC8310028

[78] Ludolf F Ramos FF Bagno FF Oliveira-da-Silva JA Reis TAR Christodoulides M. Detecting anti-SARS-CoV-2 antibodies in urine samples: A noninvasive and sensitive way to assay COVID-19 immune conversion. Sci Adv (2022) 8(19):eabn7424. doi: 10.1126/sciadv.abn7424 35559681 PMC9106288

[79] Machado JM Costa LE Dias DS Ribeiro PAF Martins VT Lage DP. Diagnostic markers selected by immunoproteomics and phage display applied for the serodiagnosis of canine leishmaniosis. Res Vet Sci (2019) 126:4–8. doi: 10.1016/j.rvsc.2019.08.010 31415928

[80] Costa MM Andrade HM Bartholomeu DC Freitas LM Pires SF Chapeaurouge AD. Analysis of leishmania chagasi by 2-d difference gel electrophoresis (2-d DIGE) and immunoproteomic: Identification of novel candidate antigens for diagnostic tests and vaccine. J Proteome Res (2011) 10(5):2172–84. doi: 10.1021/pr101286y 21355625

[81] Agallou M Athanasiou E Samiotaki M Panayotou G Karagouni E. Identification of immunoreactive leishmania infantum protein antigens to asymptomatic dog sera through combined immunoproteomics and bioinformatics analysis. PloS One (2016) 11(2):e0149894. doi: 10.1371/journal.pone.0149894 26906226 PMC4764335

[82] Costa LE Salles BCS Santos TTO Ramos FF Lima MP Lima MIS. Antigenicity of phage clones and their synthetic peptides for the serodiagnosis of canine and human visceral leishmaniasis. Microb Pathog (2017) 110:14–22. doi: 10.1016/j.micpath.2017.06.020 28629727

